# Ontogeny of audible squeaks in yellow steppe lemming *Eolagurus luteus*: trend towards shorter and low-frequency calls is reminiscent of those in ultrasonic vocalization

**DOI:** 10.1186/s40850-021-00092-8

**Published:** 2021-09-10

**Authors:** Ilya A. Volodin, Daria D. Yurlova, Olga G. Ilchenko, Elena V. Volodina

**Affiliations:** 1 Department of Vertebrate Zoology, Faculty of Biology, Lomonosov Moscow State University, Moscow, 119234 Russia; 2 Department of Behaviour and Behavioural Ecology of Mammals, A.N. Severtsov Institute of Ecology and Evolution, Russian Academy of Sciences, Moscow, 119071 Russia; 3 Department of Small Mammals, Moscow Zoo, Moscow, 123242 Russia

**Keywords:** Laboratory mammal, Discomfort calls, Vocal development, Acoustic variables, Nonlinear phenomena, Body size effect

## Abstract

**Background:**

Rodents are thought to be produced their human-audible calls (AUDs, below 20 kHz) with phonation mechanism based on vibration of the vocal folds, whereas their ultrasonic vocalizations (USVs, over 20 kHz) are produced with aerodynamic whistle mechanism. Despite of different production mechanisms, the acoustic parameters (duration and fundamental frequency) of AUDs and USVs change in the same direction along ontogeny in collared lemming *Dicrostonyx groenlandicus* and fat-tailed gerbil *Pachyuromys duprasi*. We hypothesize that this unidirectional trend of AUDs and USVs is a common rule in rodents and test whether the AUDs of yellow steppe lemmings *Eolagurus luteus* would display the same ontogenetic trajectory (towards shorter and low-frequency calls) as their USVs, studied previously in the same laboratory colony.

**Results:**

We examined for acoustic variables 1200 audible squeaks emitted during 480-s isolation-and-handling procedure by 120 individual yellow steppe lemmings (at 12 age classes from neonates to breeding adults, 10 individuals per age class, up to 10 calls per individual, each individual tested once). We found that the ontogenetic pathway of the audible squeaks, towards shorter and lower frequency calls, was the same as the pathway of USVs revealed during 120-s isolation procedure in a previous study in the same laboratory population. Developmental milestone for the appearance of mature patterns of the squeaks (coinciding with eyes opening at 9–12 days of age), was the same as previously documented for USVs. Similar with ontogeny of USVs, the chevron-like squeaks were prevalent in neonates whereas the squeaks with upward contour were prevalent after the eyes opening.

**Conclusion:**

This study confirms a hypothesis of common ontogenetic trajectory of call duration and fundamental frequency for AUDs and USVs within species in rodents. This ontogenetic trajectory is not uniform across species.

**Supplementary Information:**

The online version contains supplementary material available at 10.1186/s40850-021-00092-8.

## Background

Ontogenetic trajectories of duration and fundamental frequency (f0) in rodent ultrasonic vocalizations (USVs, over 20 kHz) may differ between species [1–7]. For example, along development from neonates to adults, the laboratory rat *Rattus norvegicus* displays the ontogenetic trajectory towards longer and lower-frequency USVs [2, 4, 5]. The ontogenetic trajectory towards shorter and higher-frequency USVs was found in fat-tailed gerbils *Pachyuromys duprasi* [8]. A different ontogenetic pattern (towards shorter and lower-frequency USVs) is characteristic of laboratory mouse *Mus musculus* [9, 10], field vole *Microtus agrestis* [11], collared lemming *Dicrostonyx groenlandicus* [12] and yellow steppe lemming *Eolagurus luteus* [7].

Ontogenetic trajectories of duration and f0 in audible calls (AUDs, below 20 kHz) may also differ between rodent species [13, 14]. For example, the audible alarm calls of speckled ground squirrels *Spermophilus suslicus* display the ontogenetic trajectory towards longer calls with a stable f0 [15]. Distinctively, yellow-bellied marmots *Marmota flaviventris* display the ontogenetic trend towards shorter and lower-frequency audible alarm calls [14, 16].

A prediction of a hypothesis that states that ontogenetic trajectories of duration and f0 are the same in AUDs and USVs within species [8] is supported by research on two rodent species: the collared lemming [12] and the fat-tailed gerbil [6, 8]. In the collared lemming, both USVs and AUDS changed to shorter and lower-frequency calls [12]. In the fat-tailed gerbil, both USVs and AUDS changed towards shorter and higher-frequency calls [6, 8].

In this study, we test this hypothesis further by studying another rodent, the yellow steppe lemming. In yellow steppe lemming, the ontogeny of acoustic parameters was only studied in detail for USVs [7], whereas the AUDs (quiet and sharp squeaks) only were described in adults and in two adolescent individuals [17]. Thus, the ontogeny of AUDs has yet to be studied in yellow steppe lemming. In case if the acoustic parameters in AUDs of yellow steppe lemming will follow the same ontogenetic trajectory as USVs, we can provide additional evidence in support of a hypothesis that the same developmental pathways of USVs and AUDs might be a common rule for rodents.

Adult and adolescent yellow steppe lemmings produce two types of audible squeaks, quiet and sharp, mainly differing in intensity and context of production [17]. In adults and adolescents, the sharp squeaks are related to handling-induced discomfort whereas the quiet squeaks are produced at a low degree of arousal [17]. Compared to quiet squeaks, the sharp squeaks of adults and adolescents are more intense, longer (0.095 vs 0.030 s), have approximately the same maximum fundamental frequency (with average maximum f0 of 1.5 kHz in adult males and of 1.7 kHz in adult females) but twice higher peak frequency and the higher power quartiles [17]. In adolescents, the f0 of the sharp squeaks is slightly higher than in adults (about 2.3 kHz) [17].

The yellow steppe lemming is a diurnal Arvicolinae rodent kept in captive populations [7, 17–19]. Physical growth and developmental milestones (e.g., eyes opening) were studied in detail in yellow steppe lemming [7]. In a captive population of Moscow Zoo, pup body mass gain from birth to 40 d of age comprises 1 g per day on average, from 6.0 ± 1.1 g in 1–4 d pups to 45.9 ± 5.6 in 37–40 d pups and to 99.0 ± 20.7 in adults [7]. Sex differences in body size lack at 25 d of age (when animals are sexed) onwards [7]. Along ontogeny, age correlates strongly positively with body mass and all linear body measurements: body length, head length, foot length and tail length. Thus, these body parameters are representative correlates of animal age [7].

In ontogeny of isolation-induced USVs from pup to adult in yellow steppe lemmings [7], the age-related growth affects the temporal and frequency acoustic parameters as well as percentages of different call contour shapes (flat, chevron, wave, upward, downward) and percentages of different kinds of nonlinear phenomena (frequency jumps, biphonations and subharmonics) [20–22]. The eyes opening at 9–12 d of age coincides with an abrupt switching of USVs to the mature pattern, including a prominent shift from chevron to upward call contour, almost complete disappearance of biphonations and the shortening of duration and decrease of f0 [7]. It has yet to be studied whether the eyes opening would coincide with an abrupt emergence of mature pattern in AUDs in yellow steppe lemmings. At the same time, adult yellow steppe lemmings primarily produced AUDs at human handling rather than at isolation [23].

The main focus of this study was on the ontogenetic changes in the acoustic parameters of the audible calls (sharp squeaks). In this study, we only analyze the sharp squeaks, as this audible call type was the most frequent call type produced at handling-induced discomfort [17, 23]. The aim of this study was to track the ontogenetic pathways of the acoustic parameters, contour shape and presence of nonlinear vocal phenomena in the audible sharp squeaks of yellow steppe lemming from birth to adulthood. In addition, we estimate the relationship between the values of the acoustic parameters and animal body size-related parameters.

## Results

### Call contours

In the total sample of 1200 sharp squeaks from the 120 subject yellow steppe lemmings belonging to 12 age classes, the most widespread was the upward contour: 417 calls (34.8%), then in order chevron contour: 379 calls (31.6%), flat: 176 calls (14.7%), complex: 171 calls (14.2%) and downward: 25 calls (4.7%).

**Fig. 1:**
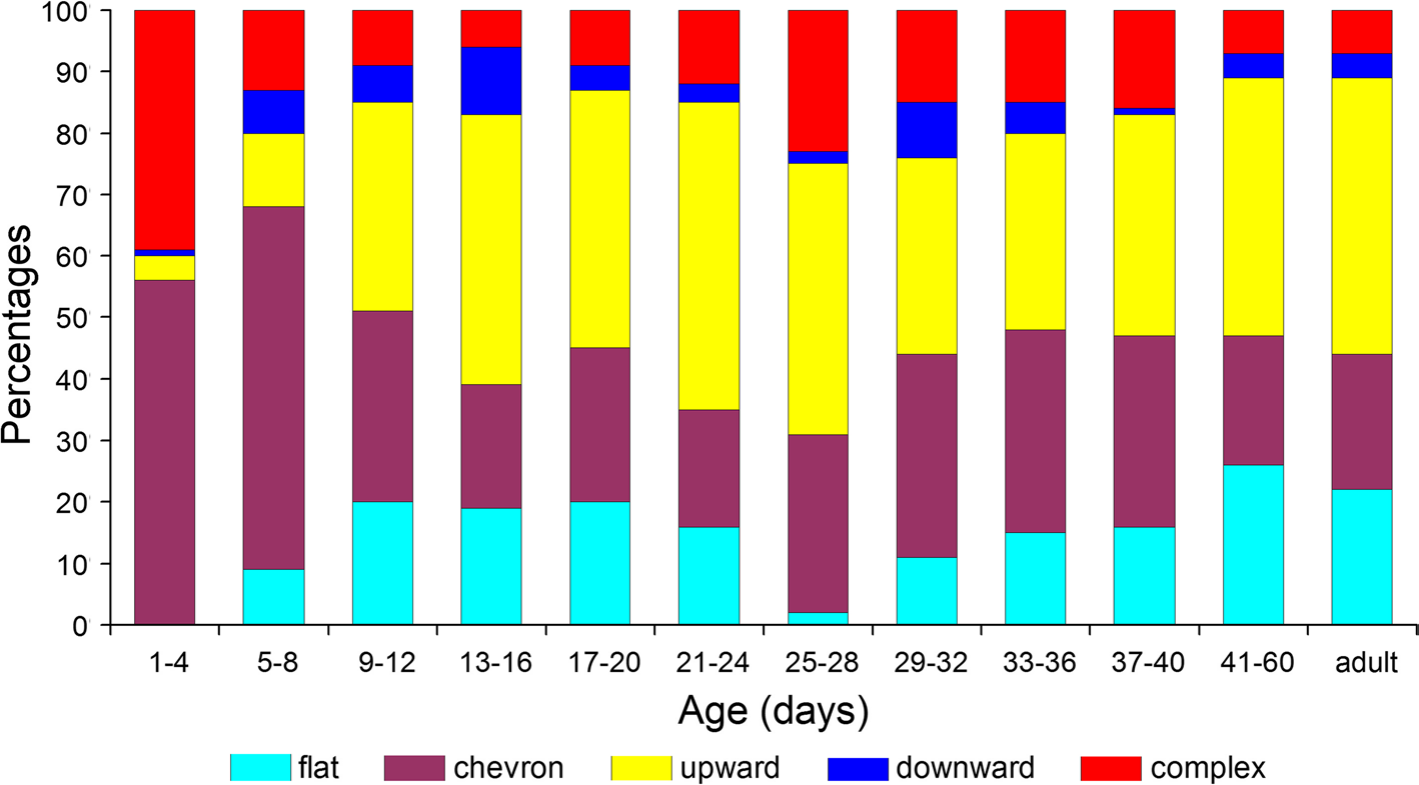
Percentages of five different contour shapes in the total sample of 1200 sharp squeaks from 120 individual yellow steppe lemmings belonging to 12 age classes

Pups at 1–4 d of age were distinctive by prevalence of the chevron (56%) and complex (39%) contours and the absence of calls with flat contour (Fig. 1). In 5–8-d pups, the chevron contour was prevailing (59% of calls), but calls with flat contour were also present (Fig. 1). In older pups, the upward contour prevailed (was present in 32–50% of calls), whereas the chevron contour (was present in 19–33% of calls) was second most common after the upward contour (Fig. 1). The age of 24–28 d is peculiar to very low number (2%) of flat contours and to a burst of complex contours (23%).

### Nonlinear phenomena

Nonlinear phenomena were detected in 329 (27.4%) of the 1200 analysed sharp squeaks. Most frequent was deterministic chaos, detected in 242 (20.2%) of the calls; subharmonics were detected in 102 (8.5%) and frequency jumps in 19 (1.6%) of the sharp squeaks. Two nonlinear phenomena were detected in 32 (2.7%) of the calls and three nonlinear phenomena were detected in one single sharp squeak.

**Fig. 2:**
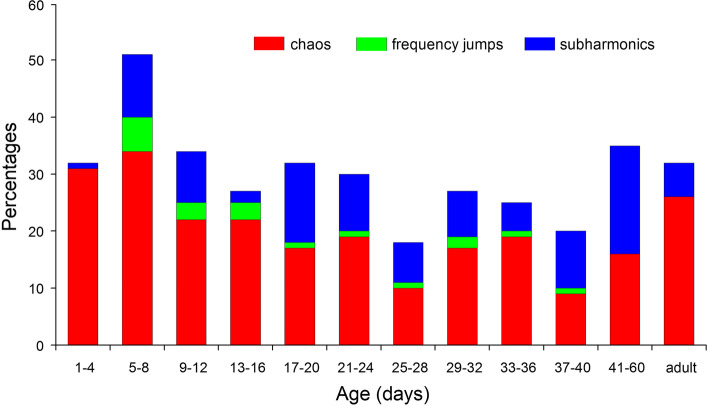
Percentages of sharp squeaks with nonlinear phenomena in the sample of 1200 sharp squeaks of 120 individual yellow steppe lemmings belonging to 12 age classes

Nonlinear phenomena occurred at any age class, being most frequent (44.0% of sharp squeaks) in 5–8-d pups and the rarest (17.0% of sharp squeaks) in 24–28-d pups. Deterministic chaos and subharmonics were detected in all age classes, whereas the frequency jumps lacked in 1–4-d pups, 41–60-d adolescents and in adults (Fig. 2).

### Age class and the acoustics of sharp squeaks

**Fig. 3:**
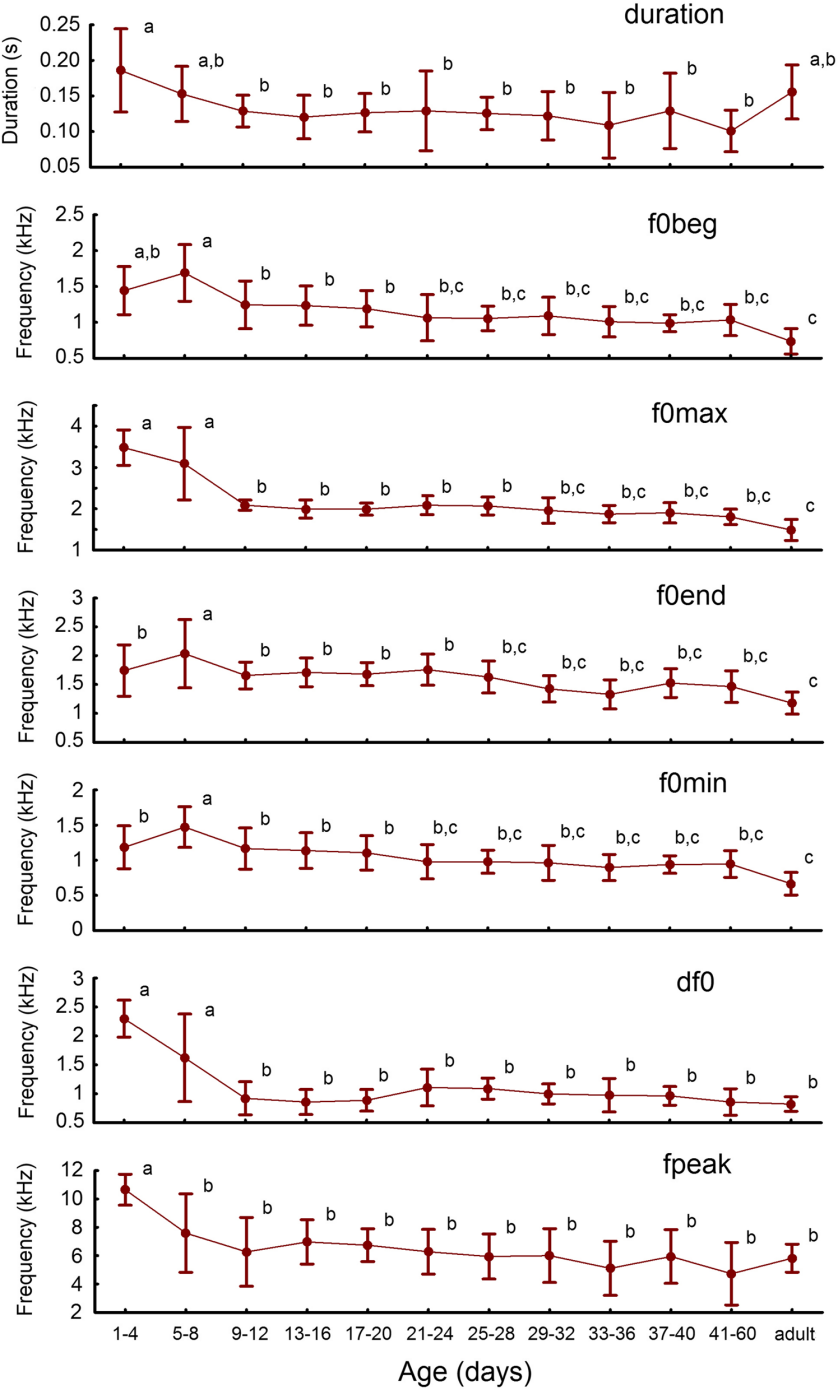
Ontogenetic trajectories for the acoustic parameters of sharp squeaks produced by 120 individual yellow steppe lemmings belonging to 12 age classes ranging from neonates to adults. *N* = 10 for each age class, the averaged values for each subject were taken. Designations: duration – call duration; f0beg – the fundamental frequency at the onset of a call; f0max – the maximum fundamental frequency; f0end – the fundamental frequency at the end of a call; f0min – the minimum fundamental frequency; df0 – the depth of frequency modulation; fpeak – the frequency of maximum amplitude; central points – means, whiskers – *SD*. The same superscripts indicate the age classes, which are non-significantly different from other age classes by the given acoustic parameter (*p* < 0.05, Tukey post hoc)

Age class significantly affected all acoustic parameters of sharp squeaks (Table 1). Duration decreased from 1–4 d to 9–12 d of age and then remained similar with those of adults. In adults, duration additionally increased compared to adolescents, although non-significantly (Fig. 3).

**Table 1:** Values (mean ± *SD*) of the acoustic parameters of sharp squeaks, produced by 120 individual yellow steppe lemmings belonging to 12 age classes and one-way ANOVA results for the effect of age class on the acoustics

Age class (days)	*n*	Duration (s)	f0beg (kHz)	f0max (kHz)	f0end (kHz)	f0min (kHz)	df0 (kHz)	fpeak (kHz)
1–4	10	0.186 ± 0.058	1.44 ± 0.34	3.48 ± 0.43	1.74 ± 0.45	1.18 ± 0.31	2.30 ± 0.32	10.65 ± 1.09
5–8	10	0.153 ± 0.039	1.69 ± 0.39	3.09 ± 0.88	2.03 ± 0.59	1.47 ± 0.29	1.62 ± 0.76	7.59 ± 2.77
9–12	10	0.129 ± 0.022	1.24 ± 0.33	2.09 ± 0.12	1.66 ± 0.23	1.17 ± 0.29	0.92 ± 0.29	6.27 ± 2.43
13–16	10	0.120 ± 0.031	1.23 ± 0.27	1.99 ± 0.22	1.71 ± 0.25	1.14 ± 0.25	0.86 ± 0.22	6.97 ± 1.56
17–20	10	0.126 ± 0.027	1.19 ± 0.25	1.99 ± 0.15	1.68 ± 0.20	1.10 ± 0.24	0.89 ± 0.19	6.74 ± 1.16
21–24	10	0.129 ± 0.056	1.06 ± 0.32	2.09 ± 0.23	1.76 ± 0.27	0.98 ± 0.24	1.11 ± 0.32	6.28 ± 1.58
25–28	10	0.125 ± 0.023	1.06 ± 0.17	2.07 ± 0.22	1.63 ± 0.28	0.98 ± 0.16	1.09 ± 0.18	5.94 ± 1.59
29–32	10	0.122 ± 0.034	1.09 ± 0.26	1.96 ± 0.31	1.42 ± 0.23	0.96 ± 0.25	1.00 ± 0.17	6.00 ± 1.90
33–36	10	0.109 ± 0.046	1.01 ± 0.21	1.87 ± 0.20	1.33 ± 0.25	0.90 ± 0.19	0.98 ± 0.29	5.11 ± 1.91
37–40	10	0.129 ± 0.053	0.99 ± 0.12	1.90 ± 0.25	1.52 ± 0.25	0.94 ± 0.12	0.96 ± 0.16	5.94 ± 1.89
41–60	10	0.101 ± 0.029	1.03 ± 0.22	1.80 ± 0.19	1.46 ± 0.27	0.95 ± 0.19	0.86 ± 0.23	4.73 ± 2.21
Adults	10	0.156 ± 0.038	0.74 ± 0.18	1.49 ± 0.26	1.18 ± 0.19	0.67 ± 0.16	0.82 ± 0.13	5.82 ± 0.99
ANOVA		*F*_11,108_ = 3.27, *p* < 0.001	*F*_11,108_ = 8.21, *p* < 0.001	*F*_11,108_ = 26.23, *p* < 0.001	*F*_11,108_ = 5.37, *p* < 0.001	*F*_11,108_ = 7.23, *p* < 0.001	*F*_11,108_ = 18.66, *p* < 0.001	*F*_11,108_ = 6.84, *p* < 0.001

Designations: *n* Number of individuals, *duration* Call duration, *f0beg* The fundamental frequency at the onset of a call, *f0max* The maximum fundamental frequency, *f0min* The minimum fundamental frequency, *f0end* The fundamental frequency at the end of a call, *df0* The depth of frequency modulation, *fpeak* The frequency of maximum amplitude

The f0max and the depth of frequency modulation decreased strongly with age, being the highest at 1–8 d of age and displaying the undistinguishable values between age classes since 9–12 d of age onwards. In adults, the f0max additionally significantly decreased compared to 9–28-d pups, whereas the depth of frequency modulation in adults remained the same as in pups (Fig. 3). The f0beg, f0end and f0min had similar ontogenetic trajectories; the 5–8-d pups were distinctive from the younger and older age classes by their highest f0 (Fig. 3). In adults, the f0beg, f0end and f0min additionally significantly lowered compared to 9–24-d pups (Fig. 3). Peak frequency was the highest at 1–4 d and did not change since 5–8 d of age onwards (Fig. 3).

### Body size and the acoustics of sharp squeaks

For calculating the body size index, we took all the five measured body size-related parameters: body mass, body, head, foot and tail lengths. All these body parameters correlated with the first PCA factor very strongly, with correlation coefficients ranging from 0.88 to 0.98 (Table 2). The first PCA factor accounted for 89.37% of variation, so we used the values of the first PCA factor as a generalizing body size index for each of the 120 subjects.

**Table 2:** Correlation coefficients between the five parameters related to body size and Principal Component Analysis (PCA) factors, eigenvalues and percent variance, described by each PCA factor

Parameter	PCA factor 1	PCA factor 2	PCA factor 3	PCA factor 4	PCA factor 5
Body mass	-0.883	0.463	-0.007	0.001	0.076
Body length	-0.981	0.103	0.004	-0.084	-0.140
Head length	-0.966	-0.105	0.178	0.156	-0.016
Foot length	-0.948	-0.264	0.093	-0.129	0.078
Tail length	-0.945	-0.168	-0.273	0.056	0.012
Eigenvalue	4.469	0.334	0.115	0.051	0.032
Percent variance	89.37%	6.67%	2.30%	1.03%	0.63%

**Fig. 4:**
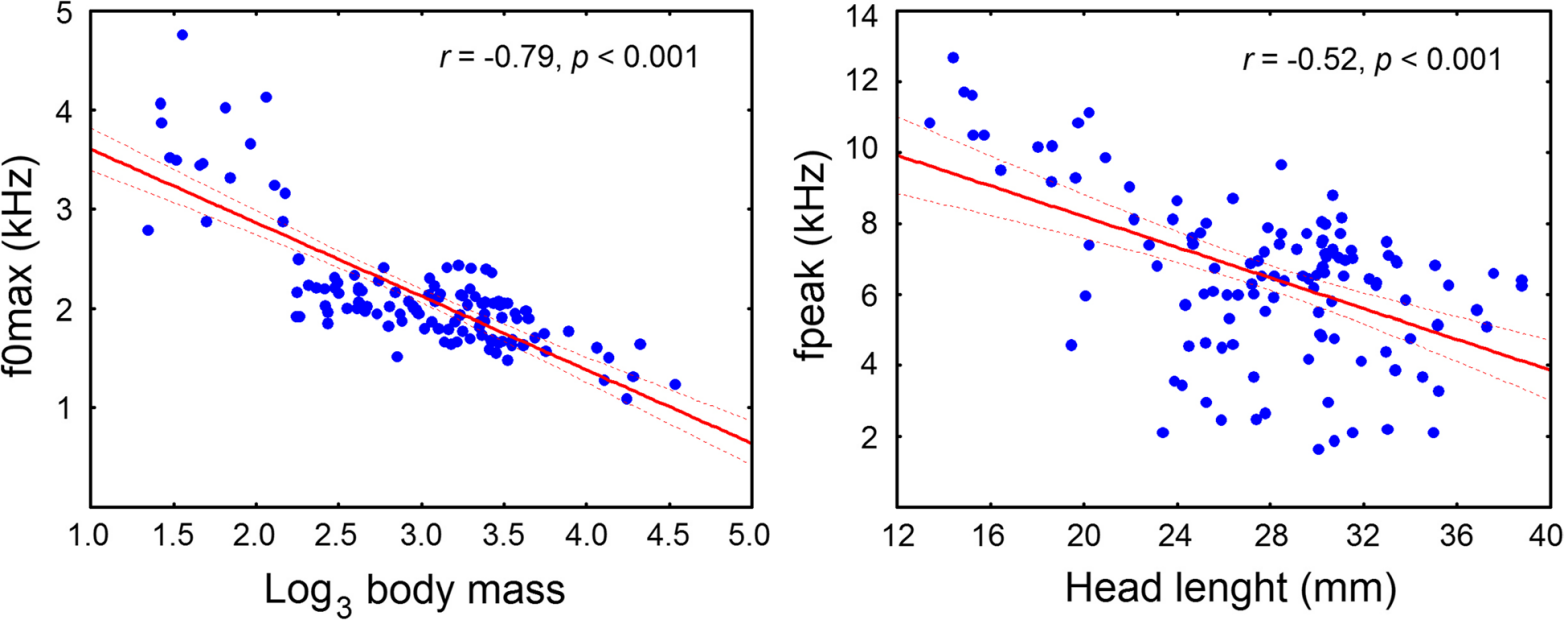
Scatterplots illustrating the relationships between the acoustic parameters of sharp squeaks and body size-related parameters of subject yellow steppe lemmings. Designations: f0max – the maximum fundamental frequency; fpeak – the frequency of maximum amplitude. Linear regression lines with 95% confidence intervals are shown

Log body mass, body length, head length, foot length, tail length and body size index (= PCA factor 1) significantly negatively correlated (after Bonferroni correction) with all acoustic parameters of sharp squeaks for the exclusion of the duration and body length (Table 3, Fig. 4). For all the five size-related parameters, the values of correlation coefficients were very similar, thus justifying that body size index is a generalizing proxy of body size in yellow steppe lemming. Therefore, the duration, peak frequency and all parameters of f0 of sharp squeaks decreased with the age-related increase of pup body size.

**Table 3:** Pearson’s correlation coefficients between body size index, log body mass, body length, head length, foot length, tail length and the acoustic parameters

Parameter	*n*	Duration	f0beg	f0max	f0end	f0min	df0	fpeak
Log_3_ body mass	120	*r* = -0.26, *p* = 0.004	*r* = -0.65, *p* < 0.001	*r* = -0.79, *p* < 0.001	*r* = -0.52, *p* < 0.001	*r* = -0.61, *p* < 0.001	*r* = -0.64, *p* < 0.001	*r* = -0.55, *p* < 0.001
Body length	120	*r* = -0.22, *p* = 0.017	*r* = -0.66, *p* < 0.001	*r* = -0.77, *p* < 0.001	*r* = -0.53, *p* < 0.001	*r* = -0.62, *p* < 0.001	*r* = -0.60, *p* < 0.001	*r* = -0.51, *p* < 0.001
Head length	120	*r* = -0.28, *p* = 0.002	*r* = -0.59, *p* < 0.001	*r* = -0.78, *p* < 0.001	*r* = -0.44, *p* < 0.001	*r* = -0.53, *p* < 0.001	*r* = -0.66, *p* < 0.001	*r* = -0.52, *p* < 0.001
Foot length	120	*r* = -0.31, *p* < 0.001	*r* = -0.62, *p* < 0.001	*r* = -0.81, *p* < 0.001	*r* = -0.47, *p* < 0.001	*r* = -0.56, *p* < 0.001	*r* = -0.69, *p* < 0.001	*r* = -0.57, *p* < 0.001
Tail length	120	*r* = -0.36, *p* < 0.001	*r* = -0.57, *p* < 0.001	*r* = -0.77, *p* < 0.001	*r* = -0.49, *p* < 0.001	*r* = -0.53, *p* < 0.001	*r* = -0.66, *p* < 0.001	*r* = -0.59, *p* < 0.001
Body size index	120	*r* = -0.25, *p* = 0.005	*r* = -0.64, *p* < 0.001	*r* = -0.79, *p* < 0.001	*r* = -0.52, *p* < 0.001	*r* = -0.60, *p* < 0.001	*r* = -0.64, *p* < 0.001	*r* = -0.54, *p* < 0.001

Threshold for significant values after Bonferroni correction comprises *p* < 0.007

Designations: *n* Number of individuals, *duration* Call duration, *f0beg* The fundamental frequency at the onset of a call, *f0max* The maximum fundamental frequency, *f0min* The minimum fundamental frequency, *f0end* The fundamental frequency at the end of a call, *df0* The depth of frequency modulation, *fpeak* The frequency of maximum amplitude

## Discussion

### Vocal ontogeny of sharp squeaks

This study provides a comprehensive analysis of the acoustic parameters of the handling-induced audible sharp squeaks in yellow steppe lemmings across age classes. We found that the sharp squeaks were produced by yellow steppe lemmings at handling from the first day of pup life and were present in all age classes and in both sexes. Sharp squeaks displayed a remarkable shift from the “juvenile” pattern (observed from the 1^st^ to the 8^th^ days of age) to the “mature” pattern (observed from the 9–12 days of age onwards). This ontogenetic shift coincided with the age of eyes opening in yellow steppe lemming [7]. After the eyes opening, pup sharp squeaks were practically undistinguishable from the sharp squeaks of their older conspecifics.

The transit from the juvenile to mature vocal pattern of sharp squeaks included: i) a prominent shift from prevalence of the chevron and complex contours to the prevalence of the upward contour; ii) the shortening of duration and the decrease of fpeak, f0 and df0. In adults, we observed a slight increase of duration and decrease of f0 (but not fpeak) compared to 1–20-d pups.

The sharp squeaks of our subject adult and 29–32-d adolescent yellow steppe lemmings were similar in their acoustic parameters to those of adult and 1-month adolescent yellow steppe lemming sharp squeaks, reported earlier [17]. They were comparable in the f0max (1.5 – 1.7 kHz in adults and 2.3 kHz subadults) and the fpeak (6.4 – 7.1 kHz in adults and 6.8 kHz in subadults) however, they displayed a larger df0 (0.4 – 0.5 kHz in adults and 0.7 kHz in adolescents). The duration of the sharp squeaks of the yellow steppe lemmings in our study was twice longer than this reported by [17] (0.073 – 0.076 s in adults and 0.052 s in adolescents) in spite of the overall similar experimental procedure in both studies. Probably, the obtained differences were account of the substantially more individuals used in our study, what could decrease the effect of individuality on the results. Nevertheless, as in the study by [17], we observed that sharp squeaks of the 29–32-d adolescents were shorter and higher in f0 but not in fpeak, although the differences did not reach the significance threshold (Table 1, Fig. 3).

### Ontogenetic pathways of sharp squeaks and USVs

The ontogenetic trajectory (towards shorter and lower-frequency calls) of the human-audible sharp squeaks, analysed in this study, was strongly reminiscent of those previously observed for the isolation-induced USVs of yellow steppe lemmings [7]. Like for the sharp squeaks, the developmental milestone for the shift to mature pattern of USVs was the age of 9–12 days (the age of eyes opening). Furthermore, similarly with sharp squeaks, the USVs of yellow steppe lemmings emerged since the 1^st^ day of pup life and occurred at all age classes and in both sexes [7]. Similar with sharp squeaks, the USVs of 1–8-d pup yellow steppe lemmings had more often the chevron contour rather than the upward contour, and had a longer duration and the higher f0 than the older animals [7]. However, whereas the occurrence of nonlinear phenomena did not change noticeably with age in the sharp squeaks, in USVs, the nonlinear phenomena were mostly detected in neonates [7]. In addition, in the sharp squeaks, the f0 slightly decreased and the duration slightly increased, whereas in USVs, the f0 and duration both decreased steadily [7].

The similarity of the ontogenetic pathways of duration and f0 between sharp squeaks and USVs in yellow steppe lemming is surprising, because rodents use different mechanisms (vocal fold vibration *vs* aerodynamic whistle) for producing respectively the audible and the ultrasonic calls [24–28]. However, especial morphological traits of the larynx enable to northern pygmy mice *Baiomys taylori* to expand their whistle USVs down to the audible range of frequencies [29]. From one side, production of both audible and ultrasonic calls is governed by breathing in the larynx and therefore potentially is not perfectly independent [30–32]. From another side, respiratory patterns can differ even between different classes of ultrasonic calls within species [33, 34], but see [25] for production of 22-kHz and 50-kHz USVs during the same expiration in laboratory rats. In addition, production of USVs in rodents is bound to sniffing [35, 36], whereas the audible vocalization putatively not.

Previously, similar ontogenetic trends of the AUDs and USVs (towards shorter and lower-frequency calls, as in the yellow steppe lemming) were found in another Arvicolinae rodent, the collared lemming [12]. Recently, similar ontogenetic trends of the AUDs and USVs (towards shorter and higher-frequency calls) were found in a Gerbillinae rodent, the fat-tailed gerbil [6, 8]. Further research is necessary to show whether similarity of ontogenetic trajectories between audible and ultrasonic calls occur in other species of rodents and whether contra-directional trends are possible for ontogeny of the audible and ultrasonic calls within species.

### Relationship of AUDs and USVs with discomfort

Contexts for eliciting USVs and AUDs were different in yellow steppe lemmings. The USVs were mostly produced at basic level of discomfort, i.e. during the experimental isolation for 2 min at unfamiliar territory [7, 23]. At the same time, the audible sharp squeaks in this study were mainly produced in more aversive context than USVs, i.e. at elevated discomfort. We therefore infer that, in yellow steppe lemming, AUDs might be triggered by the elevated level of discomfort and attend a higher animal arousal compared to USVs. Commonly, the effect of discomfort on vocal parameters has been considered either within the audible calls [37] or within the ultrasonic calls [23, 38]. However, our findings suggest that, at elevated discomfort, rodents can change the vocal production mode (from USVs to AUDs), in addition to the values of the acoustic parameters of AUDs or USVs.

### Sharp squeak acoustics and body size

In yellow steppe lemmings in this study, the f0 of sharp squeaks decreased with body growth from pups to adults. This trend followed a common rule for the AUDs of terrestrial mammals, produced with the vibration of the vocal folds: the larger an animal (and therefore the size of the vocal folds within larynx), the lower the fundamental frequency (f0) it can produce [39–41]. In many rodents, the AUDs of pups have a higher f0 than calls of adults, e.g. in the alarm calls of yellow-bellied marmots [14, 16], great gerbils *Rhombomys opimus* [42], steppe marmots *Marmota bobak* [42, 43], and in jump-yip calls of black-tailed prairie dogs *Cynomys ludovicianus* [44].

At the same time, the f0 of the audible alarm calls does not decrease from pups to adults in a few species of ground squirrels: the speckled ground squirrel, the yellow ground squirrel *Spermophilus fulvus*, the European ground squirrel *S. citelus* and the Richardson’s grounds squirrel *S. richardsonii* [13–15, 45]. In fat-tailed gerbils, the f0 of discomfort-related AUDs even increases from pups to adults [8]. The underlying morphological and/or behavioural causation of these unusual developmental trends of f0 have yet to be studied. More studies is necessary focused on studying vocal ontogeny of rodent AUDs, for better understanding of relationship between the acoustics (primarily f0) and body size.

### Acoustics of AUDs and USVs in captivity and in nature

In this study, results of similar ontogenetic trajectories of AUDs and USVs were obtained during experimental isolation and handling in captivity. There are no studies of vocalizations of yellow steppe lemmings in natural conditions. So, connection between the results reported in this study and the natural behavior of yellow steppe lemmings have yet to be investigated. Question remains, whether these call types also observed under natural circumstances such as conspecific fighting or social distress. Example of successful verifying the USVs recorded in nature with those recorded in captivity and vice versa was recently conducted for subterranean northern mole voles *Ellobius talpinus* [46]. Nevertheless, verifying bioacoustical results obtained for rodent species in laboratory and in nature is difficult because of poorly compatible behavioural contexts and occasional needs of applying in nature some experimental procedures (e.g., baiting, capturing, joining with conspecifics in a container etc.) to promote vocalizations [46]. So, “natural” calls recorded in nature are not always perfectly naturalistic. In addition, behavioural reactions of animals habituated to people to the same experimental procedure can be different in wild animals because of stronger motivation to escape than motivation to communicate vocally with a conspecific.

## Material and methods

### Study site and subjects

Audible calls (AUDs) (termed “sharp squeaks” following [17]) were collected from 120 members of a captive population of yellow steppe lemmings at Moscow Zoo, Moscow, Russia, in February-July 2018 and March–April 2020. All subjects were descendants of 7 individuals, obtained by Moscow Zoo in autumn 2016—spring 2017 from a natural population in East Kazakhstan (48^o^10’N, 84^o^25’E).

Following the methods [7, 47], before parturition, females of the captive population were checked three times per week for the appearance of a litter, and birth dates as well as the number of pups were recorded. The day of birth was considered zero day of pup life. The subjects were 110 pups from 50 litters between 1 and 60 d of age and 10 adults (7 males, 3 females) from 78 to 217 (162 ± 55) d of age. Sample of animals did not entirely overlap with those used in the previous ontogenetic study of USVs [7]: from the 120 subjects, 42 individuals were new. The adults were individually marked, whereas the small size of pups also prevented individual marking for ethical reasons until 20–25 d of age. Pups were sexed after 20–25 d of age based on visible testicles in males or vagina in females.

Study pups were offspring of 13 breeding pairs of 1–4 generation in captivity from 1 to 8 litters per pair, 3.8 ± 2.8 litters per pair on average. Litter size varied from 1 to 7 (average 3.22 ± 1.40) pups. Subjects belonged to 12 age classes: 1–4 d, 5–8 d, 9–12 d, 13–16 d, 17–20 d, 21–24 d, 28–32 d, 33–36 d, 37–40 d; 41–60 d of age and adults, 10 individuals per age class from 5–7 (5.5 ± 0.7) litters per age class, from 1 to 3 (1.88 ± 0.72) pups per litter. Each individual was tested only once, at one of 12 age classes from neonates to breeding adults; 10 individuals were tested per age class. We did not use the longitudinal approach with the same individuals repeatedly tested in each age class, because preliminary observations of zoo staff suggested that regular taking the same pups of yellow steppe lemming for weighing resulted in growth retardation of pups from the experimental litters compared to the pups not weighed [7]. Therefore, we selected to use the cross-sectional approach with many non-overlapping age classes for avoiding the effects of the repeated testing on development of the experimental pups [7].

### Animal housing

The subject animals were kept under a natural light regime at room temperature (22–25 °C), in family groups consisting of two parents and littermates of 1–3 subsequent litters. Pups at the age until 20–30 d, used in the experiments, were kept in family groups with their parents. The older pups (from 20 to 60 d) were kept with their parents, sometimes in a group could present pups of the next younger litter. Adolescents were separated from the parents at 30–60 d of age and then did not participate in experiments. The experimental adults were breeding parents of family groups. The animals were housed in wire-and-glass cages of 50 × 100x35 cm, with a bedding of sawdust of 8–10 cm and hay and various wooden shelters and cardboard pipes of 4–5 cm diameter as enrichment. They received custom-made small desert rodent chow with mineral supplements and fruits and vegetables ad libitum as a source of water.

### Experimental procedure and audio recording

All acoustic recordings were conducted in a separate room where no other animals were present, at room temperature 22–25 °C during daytime, at the same level of background noise. For recording the AUDs (sampling rate 48 kHz, 16 bit resolution) we used a solid state recorder Marantz PMD-660 (D&M Professional, Kanagawa, Japan) with a Sennheiser K6-ME66 cardioid electret condenser microphone (Sennheiser electronic, Wedemark, Germany), flat frequency response from 0.04 to 20 kHz. The microphone was established stationary at distance 35 cm above the animal. The obtained recordings had a high signal/noise ratio, the reverberation practically lacked. Recording of each trial was stored as a wav-file.

Each subject was tested singly only in one experimental trial. Immediately before a trial, the focal animal was taken from the home cage and transferred in a small clean plastic hutch to the experimental room within the same floor of the building. Time from removal of the focal animal from the cage to the start of an experimental trial did not exceed 60 s. During the trial, the animal was isolated in an experimental setup, either clean plastic hutch (190 × 130x70 mm for 1–12 d pups) or in a plastic cylinder without bottom (diameter 193 mm, high 170 mm for 13–60 d pups and adults), placed on even plastic table surface. Both the plastic huge and cylinder were open from above, i.e. from the side where the microphone was placed. The recording started, when the focal animal was placed to the experimental setup. Each trial took place in four stages: the isolation stage (120 s); the touch stage (120 s), the handling stage (120 s) and the measurement stage (120 s). Aside isolation, the focal pups experienced also a cooling, due to the imperfect thermoregulation of 1–12 d pups with still poorly developed fur cover.

For the duration of the isolation stage, a focal animal was located either in a plastic hutch or cylinder. For the duration of the touch stage, the experimenter (DDY or IAV) gently touched the focal animal with a cotton bud, approximately two times per second. For the duration of the handling stage, the experimenter took the focal animal in hands and rotated it on its back following [48]. For the duration of the measurement stage, the experimenter measured body length, head length, foot length and tail length with an electronic caliper (Kraf Tool Co., Lenexa, Kansas, US, accurate to 0.01 mm), continuing keeping animal in hands. We measured body length from the tip of the snout to the anus, and head length from the tip of the snout to the occiput. We measured foot length from the heel to the tip of the middle toe, and tail length from anus to the tip of the tail. These measurements were repeated thrice and the mean value was taken for analysis. The end of measurements was the end of the trial. After the trial, the focal animal was weighed on G&G TS-100 electronic scales (G&G GmbH, Neuss, Germany, accurate to 0.01 g). Weighing was done in the same plastic hutch which was used for transferring the animal to the experimental setup. The body size-related parameters and body mass were used as proxies of body size for further comparison with the acoustic parameters of the audible calls.

If more than one littermate per litter was tested, after the end of a trial, the focal pup was placed to a heating hutch with a bedding of a cotton fabric, standing in the neighboring room. Trials with all focal littermates were done consequently in the same manner. Then all of them were simultaneously returned to their home cage to their parents; the time of pup stay out of the nest did not exceed 30 min. The adults were taken from their home cages before experiments with a clean plastic glass and returned to the cage after the test trial. The experimental setup was rubbed with napkin wetted with alcohol after each experimental trial, to avoid effect of smell on vocal behaviour of the next focal animal in the next trial [35].

### Call samples

Using visual inspection of spectrograms of acoustic files created with Avisoft SASLab Pro software (Avisoft Bioacoustics, Berlin, Germany) we classified the audible squeaks to quiet and sharp ones based on their relative intensity and spectrographic pattern following [17]. We selected for analysis 10 sharp squeaks per individual recorded at the experimental stages 3 and 4 (i.e. at the stages of handling, where the sharp squeaks were available most frequently), randomly among those considered eligible, of high sound-to-noise ratio and without superimposed noise, from different parts of each recording, avoiding taking calls following each other. Call contour and presence of nonlinear phenomena were not considered as selection criteria. We considered vocal emissions as separate calls whenever they were separated from each other with interval over 20 ms. In total, from 120 individuals at 12 age classes, we selected for acoustic analyses 1200 sharp squeaks.

### Acoustic analysis

Measurements of acoustic parameters of sharp squeaks have been conducted with Avisoft and exported to Microsoft Excel (Microsoft Corp., Redmond, WA, USA). As minimum fundamental frequency of sharp squeaks always exceeded 0.1 kHz, before measurements all wav-files were subjected to 0.1 kHz high-pass filtering, to remove low-frequency noise.

**Fig. 5:**
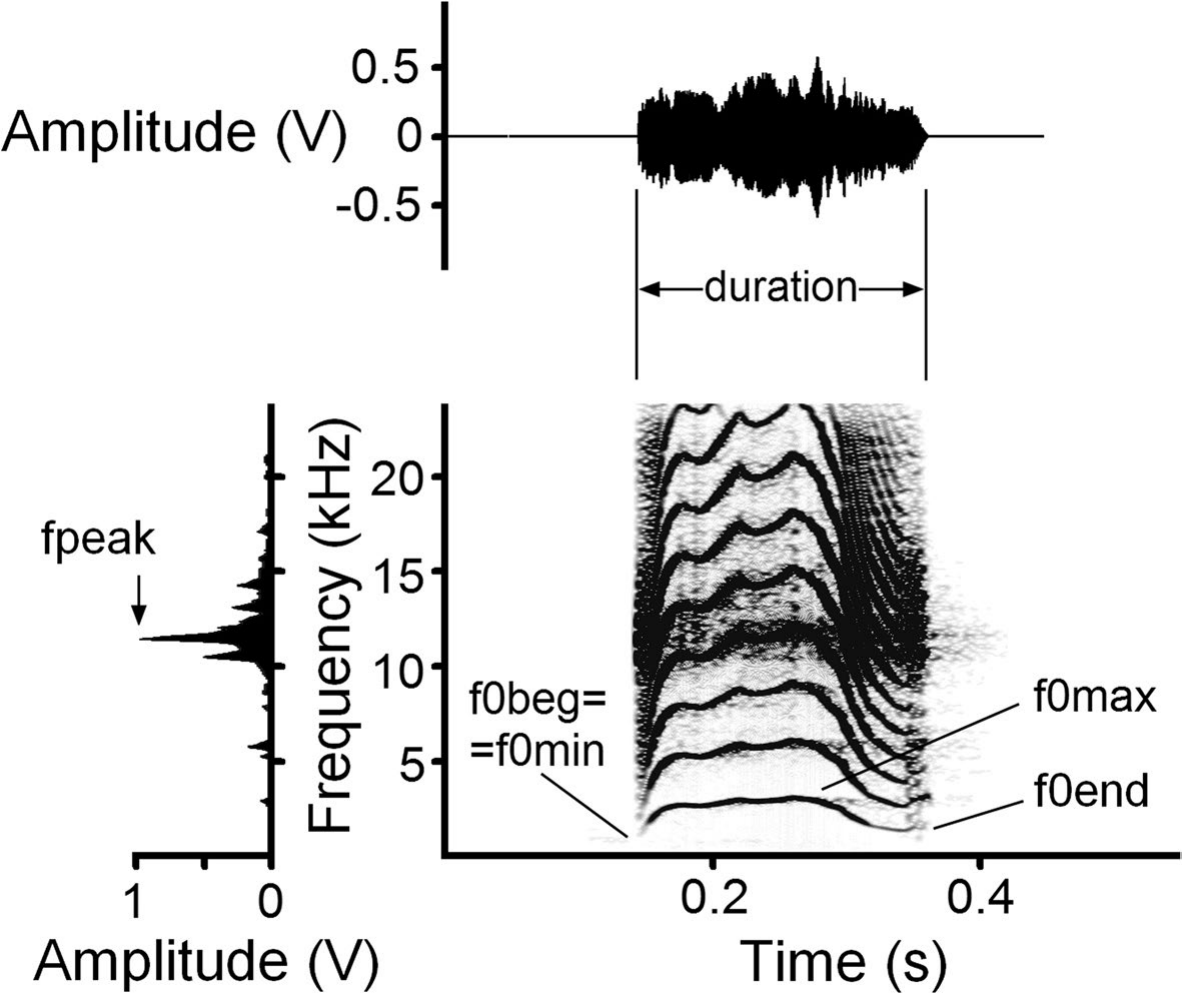
Measured parameters for sharp squeaks exemplified by 4-day old pup sharp squeak with chevron contour. Spectrogram (right), mean power spectrum of the entire call (left) and wave-form (above). Designations: duration – call duration; f0max – the maximum fundamental frequency; f0min – the minimum fundamental frequency; f0beg – the fundamental frequency at the onset of a call; f0end – the fundamental frequency at the end of a call; fpeak – the frequency of maximum amplitude. Spectrogram was created using sampling frequency 48 kHz, a Hamming window, Fast Fourier Transform (FFT) 1024 points, frame 50% and overlap 96.87%

For each sharp squeak, we measured, in the spectrogram window of Avisoft (sampling frequency 48 kHz, a Hamming window, Fast Fourier Transform (FFT) 1024 points, frame 50%, overlap 93.75%, providing frequency resolution 47 Hz and time resolution 1.33 ms), the duration with the standard marker cursor and the maximum fundamental frequency (f0max), the minimum fundamental frequency (f0min), the fundamental frequency at the onset of a call (f0beg), and the fundamental frequency at the end of a call (f0end) with the reticule cursor (Fig. 5 and Table S1). We calculated the depth of frequency modulation (df0) as the difference between f0max and f0min. For each sharp squeak, we measured, in the power spectrum window of Avisoft, the frequency of maximum amplitude (fpeak) from the call’s mean power spectrum (Fig. 5 and Table S1).

### Call contours and nonlinear phenomena

**Fig. 6:**
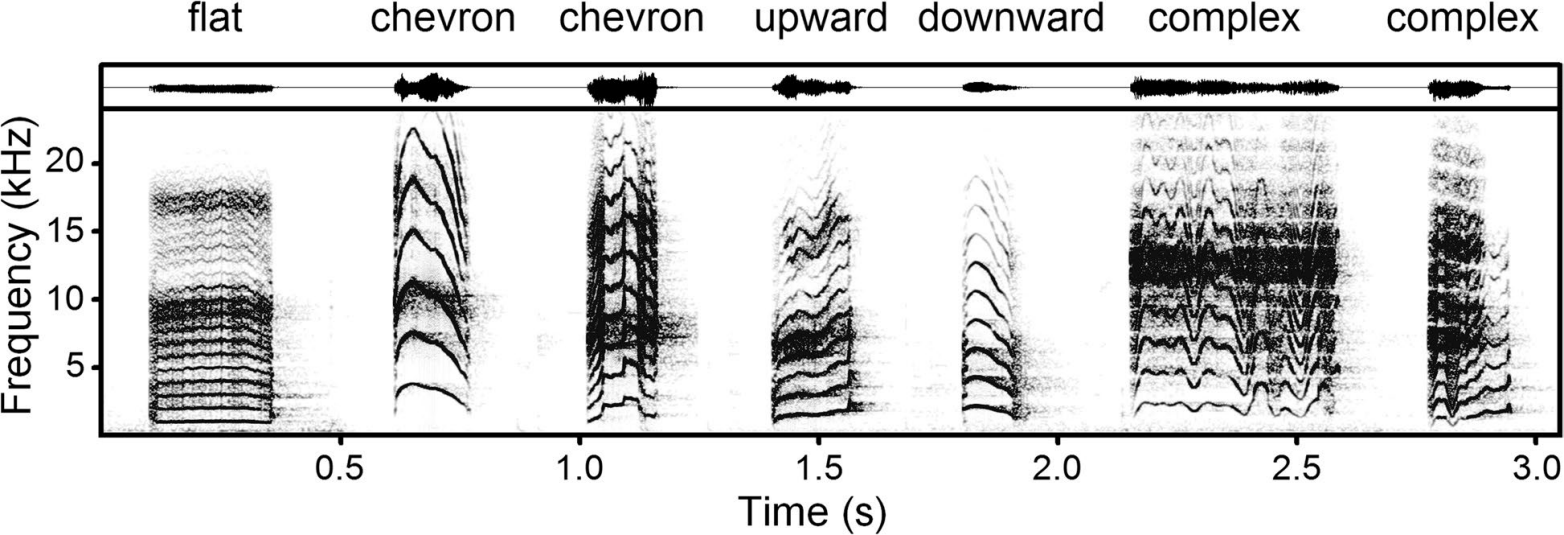
Five contour shapes occurring in sharp squeaks of pup and adult yellow steppe lemmings: flat from 14-day old pup; chevron from 5-day old pup; chevron from 29-day old pup; upward from 37-day old pup; downward from 8-day old pup; complex from 2-day old pup; complex from 28-day old pup. The Audio file is available at Audio S2. Spectrogram was created using sampling frequency 48 kHz, a Hamming window, FFT 1024 points, frame 50% and overlap 93.75%

In the spectrogram window of Avisoft, we classified the sharp squeaks manually accordingly to the five contour shapes: flat, chevron, upward, downward and complex, following categorization developed for USVs of yellow steppe lemmings [7] (Fig. 6 and Audio S2). Flat contour was denoted when the difference between f0min and f0max was less than 0.6 kHz. When the difference between f0min and f0max exceeded 0.6 kHz, the denoted contour could be the chevron (up-down one time), upward (ascending from start to end), downward (descending from start to end) or complex (up-down many times or U-shaped).

**Fig. 7:**
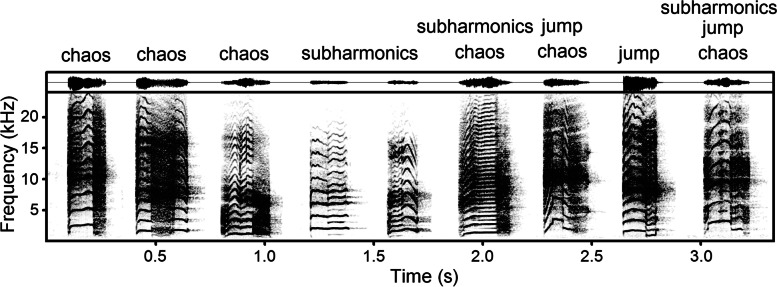
Nonlinear phenomena occurring in the sharp squeaks of pup and adult yellow steppe lemmings: deterministic chaos from a 5-day old pup; chaos from a 15-day old pup; chaos from a 35-day old pup; subharmonics from a 28-day old pup; subharmonics from a 25-day old pup; subharmonics and chaos from an adult male; frequency jump and chaos from a 6-day old pup; frequency jump from a 15-day old pup; subharmonics, frequency jump and chaos from a 6-day old pup. The Audio file is available at Audio S3. Spectrogram was created using sampling frequency 48 kHz, a Hamming window, FFT 1024 points, frame 50% and overlap 93.75%

For each sharp squeak, we noted the presence of nonlinear phenomena (Fig. 7 and Audio S3): frequency jumps, deterministic chaos and subharmonics [20–22]. Frequency jump was denoted when f0 suddenly changed for ≥ 1 kHz up or down [20–22]. Deterministic chaos was denoted when the chaotic segments were found in call spectra, these chaotic segments could contain residual harmonic structures within the chaotic episodes [20–22] (Fig. 7). Subharmonics were denoted when the intermediate frequency bands of 1/2 or 1/3 of f0 were present between harmonics (Fig. 7). We considered that the given nonlinear phenomenon was present in call spectrum, if it occupied 10% or more of the entire call duration. For calls with frequency jumps, we identified the contour shape by virtual smoothing the contour as if frequency jump was lacking and the fundamental frequency contour was continuous, following [7].

### Statistical analyses

Statistical analyses were made with STATISTICA, v. 8.0 (StatSoft, Tulsa, OK, USA), all means are given as Mean ± *SD*. Significance levels were set at 0.05, and two-tailed probability values are reported. For each subject, the averaged values of each acoustic parameter over 10 calls (sharp squeaks) were used for the statistical comparisons (Table S1), to decrease the number of degrees of freedom for more robust results. Distributions of all acoustic and body parameter values did not depart from normality (Kolmogorov–Smirnov test, *p* > 0.05).

We used a one-way ANOVA with Tukey HSD (Honestly Significant Difference) test to estimate the effects of age on the acoustics. We used Principal Component Analysis (PCA) to estimate the degrees of correlation between the five parameters related to body size, and for calculating body size index based on these parameters. We used a Pearson correlation with Bonferroni correction to estimate potential correlation between the parameters related to body size, the body size index and the acoustic parameters.

## Supplementary Information

**Additional file 1: Table S1.** Mean values for acoustic variables of AUD calls, body weight, body length, head length, foot length, tail length and body size index for 120 individual pup and adult yellow steppe lemmings.

**Additional file 2: Audio S2.** AUD calls of yellow steppe lemmings exemplifying the five contour shapes. AUD with contour flat from 14-day old pup; AUD with contour chevron from 5-day old pup; AUD with contour chevron from 29-day old pup; AUD with contour upward from 37-day old pup; AUD with contour downward from 8-day old pup; AUD with contour complex from 2-day old pup; AUD with contour complex from 28-day old pup. Order as on Fig. 6. Sampling frequency of the acoustic file is 48 kHz.

**Additional file 3: Audio S3.** AUD calls of yellow steppe lemmings exemplifying the three kinds of nonlinear phenomena. AUD call with deterministic chaos from a 5-day old pup; AUD call with chaos from a 15-day old pup; AUD call with chaos from a 35-day old pup; AUD call with subharmonics from a 28-day old pup; AUD call with subharmonics from a 25-day old pup; AUD call with subharmonics and chaos from an adult male; AUD call with frequency jump and chaos from a 6-day old pup; AUD call with frequency jump from a 15 day old pup; AUD call with subharmonics, frequency jump and chaos from a 6-day old pup. Order as on Fig. 7. Sampling frequency of the acoustic file is 48 kHz.

## Data Availability

All data are presented in electronic supplementary material.

## Abbreviations

AUD: Audible vocalization
df0: The depth of frequency modulation
f0beg: The fundamental frequency at the onset of a call
f0end: The fundamental frequency at the end of a call
f0max: The maximum fundamental frequency
f0min: The minimum fundamental frequency
FFT: Fast Fourier Transform
fpeak: The frequency of maximum amplitude
PCA: Principal Component Analysis
USV: Ultrasonic vocalization
